# Modernized teaching material and methodology for road safety educators 

**Published:** 2019-08

**Authors:** Hele Leek-Ambur, Anu Kikas

**Affiliations:** ^*a*^Foundation Läänemaa/Tallinn University Haapsalu College, Estonia.; ^*b*^Foundation Läänemaa, Estonia.

## Abstract

One of the project main objectives was to develop new study material for road safety education providers in order to improve road safety through the development of driving teachers' education in Estonia, Finland and Norway. In order to complete this objective several activities were carried out: 1. Four international project meetings - Originally were planned three meetings each connected with one of the other activities (two with student mobilities and one with dissemination event), but for the compilation of a book the writers needed one additional meeting, just to clarify different aspects concerning the textbook. 2. Two student teaching and learning mobilities - one in Finland (participated: 5 students from Estonia, 5 students from Norway and 8 students from Finland) and the other in Norway (participated: 6 students from Estonia, 9 students from Norway and 4 students from Finland). Both student mobility’s contributed into the writing of a textbook. In the first mobility students and teachers tested several ideas and gather thoughts about topics that should be in the textbook and in second mobility students gave feedback to the articles composed. 3. Dissemination event in Estonia - road safety conference: Theory and Practice in Driver Education. The dissemination event was dedicated to the intellectual output and was a success in every way. There were two keynote speakers talking about overall ideas and state of road safety in Europe and also all the writers introducing their chapter of the textbook. All the participants (119 people) had a chance to acquire the new textbook. The textbook was released in October 2018 and all the project partners had 200 copies of the book. As the book was written in English TUHC translated all the articles into Estonian and the articles together with the videos made from the conference are now being used as a study material in Traffic safety curriculum. The created textbook is also available for all the interested parties on project partners’ websites. During the project all the participating teachers and students gained new experience, improved their English language skills, improved cooperation and intercultural skills. Project partners gained new teaching and learning material, strengthened their international cooperation and improved the quality of higher education in their institutions.

**Keywords:**

Pedagogical Challenges of a Modern Driving Teacher; Active Study Methods in Teaching Drivers of Motor Vehicles; Responsible Driver; Impulse Awareness

